# Surgical antimicrobial prophylaxis in open reduction internal fixation procedures at a metropolitan hospital in Australia: a retrospective audit

**DOI:** 10.1186/s12893-021-01398-7

**Published:** 2021-11-23

**Authors:** Sarah Hassan, Vincent Chan, Julie Stevens, Ieva Stupans, Juliette Gentle

**Affiliations:** 1 Pharmacy, School of Health and Biomedical Sciences, RMIT University, Bundoora, VIC Australia; 2 Clinical and Health Sciences, University of South Australia, Adelaide, SA Australia; 3 Adelaide Medical School, Faculty of Health and Medical Sciences, University of Adelaide, Adelaide, SA Australia; 4 Department of Orthopaedics, Northern Health, Epping, VIC Australia

**Keywords:** Open reduction internal fixation, Closed fractures, Surgical antimicrobial prophylaxis, Guideline adherence, Guideline update

## Abstract

**Background:**

Open reduction internal fixation (ORIF) of closed fractures is a required indication for surgical antimicrobial prophylaxis (SAP). Guidelines contain recommendations on how best to prescribe SAP, however, adherence to SAP guidelines remains suboptimal. The Australian *Therapeutic Guidelines: Antibiotic* v16 (updated April 2019) advocates for single dose prophylaxis for ORIF procedures. There is a paucity of information on how SAP is prescribed for ORIF of closed fractures in Australian hospitals. The aim of this study was to identify prescribing practice and to evaluate guideline adherence pre- and post-guideline update.

**Methods:**

A retrospective audit was conducted for patients undergoing an ORIF of closed fractures at a metropolitan teaching hospital in a 6-month period during 2018 (pre-guideline update) and 2019 (post-guideline update). Data were collected on prescribing practice (perioperative antibiotics prescribed, dose, time and route of administration and duration of prophylaxis) and compared to SAP recommendations in *Therapeutic Guidelines: Antibiotic* v15 (2018) and v16 (2019). Descriptive statistics and Chi square tests were used to report categorical variables. Binary logistic regression was used to identify factors associated with guideline adherence. A p-value < 0.05 was deemed statistically significant.

**Results:**

Data were collected for a total of 390 patients (n = 185, 2018; n = 205, 2019). Cefazolin was the most commonly prescribed antibiotic as per guideline recommendations, with variable, yet appropriate doses observed across the two audit periods. While 78.3% of patients received SAP for the correct duration in 2018, only 20.4% of patients received single dose prophylaxis in 2019. Overall adherence to guidelines was 63.2% in the 2018, and 18.0% in the 2019 audit periods respectively. Patient age was significantly associated with an increase in overall guideline adherence, while lower limb fractures, an American Society of Anesthesiologists (ASA) score of 3 and emergency admissions were associated with decreased overall adherence to SAP guidelines.

**Conclusion:**

Adherence to guidelines was greater with v15 (2018) compared with v16 (2019). Patient factors, including limb fracture site and ASA score, had little impact on guideline adherence. Further research is required to understand what influences guideline adherence in the orthopaedic setting.

**Supplementary Information:**

The online version contains supplementary material available at 10.1186/s12893-021-01398-7.

## Introduction

Surgical antimicrobial prophylaxis (SAP) accounts for one in six antibiotic prescriptions in hospitals worldwide [1], with 14% of all antimicrobials prescribed in Australian hospitals for SAP [2]. Inappropriate prescribing and poor adherence to SAP guidelines has been noted across all surgical disciplines, including orthopaedic surgery [3–5]. The inappropriate use of SAP has multiple consequences, including the development of antimicrobial resistance, an increase in adverse events, length of hospital stay as well as costs to the healthcare system [6].

SAP recommendations are often presented in the form of clinical practice guidelines, with suggestions on the appropriate prescribing and administration of antibiotics. Optimal SAP is dependent on fulfilment of the following key quality indicators: selection of correct antimicrobial for indication; administration of correct dose via correct route; administration of preoperative antibiotics at the correct time with intraoperative doses given at the correct interval and administration of SAP for the recommended duration [7, 8].

Open reduction internal fixation (ORIF) procedures are a widely performed orthopaedic procedure, however, substantial variability exists for the choice, dose and regimen of prophylactic antibiotics. They are classified as clean procedures, with an estimated surgical site infection (SSI) rate of 1–5% [9]. Recent guidelines from the Centers for Disease Control and Prevention (CDC) and the World Health Organisation (WHO) [10–12] have suggested the use of a single preoperative antibiotic dose is sufficient for most clean procedures, including that of internal fixations. Single dose prophylaxis often refers to the use of a single dose of preoperative antibiotics, including any intraoperative dose that may be administered (dependent on procedure duration and half-life of administered antibiotic) [13].

In Australia, recommendations for SAP prescribing in orthopaedic surgery can be found in a national guideline known as the *Therapeutic Guidelines: Antibiotic* [14]*.* Guidelines advocate for 2 g of cefazolin to be administered within 60 min before surgical incision (or 3 g for patients above 120 kg). For patients with an immediate severe or delayed severe hypersensitivity to penicillins, the use of vancomycin (15 mg/kg) within 120 min before surgical incision is recommended, with the use of both cefazolin and vancomycin recommended for patients colonised or infected with methicillin-resistant *Staphylococcus aureus* (MRSA) [14].

*Therapeutic Guidelines: Antibiotic* was updated in April 2019 from version 15 to version 16. The key difference between the previous (v15) and current (v16) versions relate to the recommended duration of prophylaxis for most orthopaedic procedures (including internal fixations). Whilst *Therapeutic Guidelines: Antibiotic* version 15 mentions there is little evidence to support the use of SAP for greater than 24 h after induction of anaesthesia, version 16 states that SAP should be administered as a single preoperative dose, with no further doses once surgery is complete, aligning with guideline recommendations from both the CDC and WHO.

Despite the recommendations in clinical practice guidelines and their widespread availability, it has been noted that adherence to SAP guidelines is suboptimal [15–17]. Non-concordant SAP prescribing can be a result of failing to comply with any of the given key quality indicators, with antibiotics administered at the incorrect time or for a prolonged duration often being the cause [4, 15, 16, 18, 19].

The *Therapeutic Guidelines: Antibiotic* is widely accessible in Australian hospitals [20, 21] and provides national guidance on antimicrobial use in Australia [20]. Locally endorsed guidelines can also be used to guide practice and are local adaptations of the *Therapeutic Guidelines* based on antimicrobial resistance trends or special patient populations [20]. While multiple global studies have identified how SAP is prescribed for various surgical procedures and the adherence rates to SAP guidelines, little is known about the prescribing practice of antibiotics for ORIF of closed fractures in Australian hospitals. Adherence rates to the *Therapeutic Guidelines: Antibiotic* is also unknown, and it is unclear whether adherence to guidelines is greater following guideline update in this setting. Thus, the aim of this study was to determine how SAP is prescribed for ORIF of closed fractures at a metropolitan hospital in Melbourne, Australia and to determine how practice compares to guideline recommendations prior to, and following, guideline update.

## Methods

### Study setting and design

A retrospective audit was conducted for patients admitted for an ORIF procedure at Northern Health, a major tertiary health service located in Melbourne, Australia. Patients who were admitted for either an elective or emergency ORIF procedure were reviewed during one of two specified 6-month periods, the first audit in 2018 and the second audit in 2019. This study was approved as a Quality Improvement and Innovation project (ALR26.2019) by the Northern Health Office of Research, Ethics and Governance.

### Inclusion and exclusion criteria

Patients were included if they underwent either an emergency or elective ORIF of closed fractures of the upper and lower limbs between January and June 2018 or July and December 2019. This time frame was chosen as guidelines were updated in April 2019, thus providing an opportunity to determine practice prior to, and after, guideline update. Procedures were categorised as elective if a patient had a planned admission, rather than emergency admission, to theatre.

The focus was on procedures that contained a closed fracture of the upper limbs (clavicle, humerus, radius and ulna) and lower limbs (ankle, femur, neck of femur and tibia) as fractures at these sites are most commonly operated on at Northern Health. Patients were excluded if they presented with an open fracture, underwent a revision ORIF or were being treated with antibiotics for a pre-existing infection.

### Data collection

A list of patients who underwent an ORIF procedure during the defined time periods was obtained from the hospital’s Health Information Service, with each record reviewed to determine eligibility for inclusion. Data were collected by reviewing medical records in the hospital’s Digital Patient Chart system (Clinical Patient Folder), with data obtained from the emergency department record, anaesthetic record sheet, operation sheet, medication chart, inpatient progress notes as well as outpatient notes. Patient laboratory data were accessed to record preoperative renal function where available.

A standardised data collection tool was used, with the following parameters collected: demographics [age, gender, allergy status, comorbidities, smoking status, length of stay (LOS), body mass index (BMI; where height and weight were available)], renal function, American Society of Anesthesiologists (ASA) score, type of admission, duration of procedure, fracture site, perioperative prescribing regimen (whether preoperative antibiotics were given, drug administered, time and route of administration, postoperative orders, duration of prophylaxis from induction), whether an SSI developed and if so, its management.

Overall prescribing was deemed compliant if each of the following key quality indicators matched guideline recommendations: antimicrobial choice, dose, route of administration and duration of prophylaxis. If at least one of the key quality indicators was non-compliant with guidelines, then prescribing was deemed non-compliant overall. Overall compliance was classified as unknown if data were missing or unavailable for the quality indicators mentioned above.

Dose prescribed was considered appropriate if it differed from the recommended 2 g of cefazolin but took into account a patient’s age, weight or renal function. Results were compared to recommendations in the *Therapeutic Guidelines: Antibiotics* version 15 and 16; the first audit period compared to version 15 and the second audit period to version 16. The two audit periods were also compared against each other to determine level of adherence pre- and post-guideline update.

### Statistical analyses

Data were recorded in Microsoft Excel and subsequently analysed using SPSS Version 26 (IBM Inc., Chicago, IL). Descriptive statistics were used to report categorical variables through percentages and frequencies, including the proportion of patients who received pre-, intra- and postoperative antibiotics, the number of doses administered on the ward, postoperative antibiotic instructions listed by surgeons, the duration of prophylaxis from time of induction and the proportion of patients who developed an SSI. Chi square tests were used to determine associations between categorical variables, and to compare overall compliance between the two audit periods.

A binary logistic regression model was used to determine whether any factors were associated with overall adherence to each version of the guidelines, and in particular to dose and duration of SAP. The following variables were included in the analysis: gender, age group, ASA score, limb fracture site, type of admission, LOS and whether a patient had diabetes. Odds ratios, 95% confidence intervals and p-values are reported for the results of the binary logistic regression. A p-value < 0.05 was considered statistically significant for all results.

## Results

Data were collected for a total of 390 patients (185 patients and 205 patients in the 2018 and 2019 audit period, respectively). Overall compliance with guideline recommendations was 63.2% for the 2018 audit period and only 18.0% for the 2019 audit period. A summary of patient demographics can be found in Table 1.

**Table 1:** Summary of patient demographics

	2018 (n = 185)	2019 (n = 205)
Gender—n (%)		
Male	84 (45.4%)	93 (45.4%)
Female	101 (54.6%)	112 (54.6%)
Mean age (years) ± SD	58.4 ± 26.7	53.2 ± 24.8
LOS (days)—n (%)		
0–3	93 (50.3%)	110 (53.7%)
> 3	92 (49.7%)	95 (46.3%)
ASA score—n (%)		
1	43 (23.2%)	50 (24.4%)
2	67 (36.2%)	72 (35.1%)
3	44 (23.8%)	48 (23.4%)
4	7 (3.8%)	9 (4.4%)
5	0 (0%)	1 (0.5%)
Unknown	24 (13.0%)	25 (12.2%)
Type of admission—n (%)		
Elective	66 (35.7%)	77 (37.6%)
Emergency	119 (64.3%)	128 (62.4%)
Fracture site—n (%)		
Upper limbs	61 (33.0%)	63 (30.7%)
Clavicle	4 (6.6%)	11 (17.5%)
Radius	45 (73.8%)	40 (63.5%)
Ulna	2 (3.3%)	1 (1.6%)
Multiple sites	9 (14.7%)	10 (15.9%)
Other	1 (1.6%)	1 (1.6%)
Lower limbs	124 (67.0%)	142 (69.3%)
Ankle	39 (31.5%)	61 (43.0%)
Femur	17 (13.7%)	26 (18.3%)
Hip (Neck of femur)	63 (50.8%)	50 (35.2%)
Multiple sites	5 (4.0%)	3 (2.1%)
Other	0 (0%)	2 (1.4%)

### Antibiotic prescribing regimen

#### Preoperative antibiotics

Almost all patients in both audit periods had antibiotics administered preoperatively (97.3% and 95.6%, respectively). Cefazolin was the most commonly prescribed and administered antibiotic, and clindamycin was prescribed and administered for patients with a penicillin allergy. Preoperative antibiotic doses prescribed ranged from 400 mg to 3 g in the 2018 audit period and 600 mg to 3 g in the 2019 audit period.

#### Postoperative antibiotics

In the 2018 audit period, postoperative instructions were indicated on the operation sheet in an inconsistent manner, with 24 h of intravenous (IV) antibiotics being the most common instruction (55.7%). Seventeen patients (9.2%) did not have an instruction as to whether postoperative antibiotics were to be administered. Almost all patients (93.5%) received antibiotics postoperatively on the ward, with 90.3% of patients receiving cefazolin. The number of postoperative doses administered varied from zero to nine doses, with approximately half of the patients (49.2%) receiving three doses. Three postoperative doses were most commonly observed in patients aged over 80 years (36.2%). Antibiotics were administered intravenously in both the preoperative and postoperative setting.

For the 2019 study period, instructions for postoperative antibiotics varied from no further requirement for antibiotics to 24 h of IV antibiotics, with two postoperative doses being the most common instruction (23.9%). Forty-seven patients (22.9%) did not have an instruction recorded for whether postoperative antibiotics were to be administered.

Cefazolin was prescribed and administered postoperatively in 78.5% of patients. Only 20.4% of patients did not receive postoperative antibiotics as per guideline recommendations. The number of postoperative doses administered ranged from zero to four doses with just over half of patients receiving two doses (51.7%). Single dose prophylaxis (i.e. zero postoperative doses) was most commonly observed in patients aged 21–40 years (45.2%).

All antibiotics were administered intravenously in the preoperative, intraoperative and postoperative setting. Results of compliance with *Therapeutic Guidelines: Antibiotics* version 15 and 16 recommendations can be found in Table 2.

**Table 2:** Compliance with *Therapeutic Guidelines: Antibiotics* recommendations

Summary of Therapeutic Guidelines recommendations	Compliance with version 15 guidelines (2018)	Compliance with version 16 guidelines (2019)
All patients should receive preoperative antibiotics for internal fixations	97.3% (180/185)	95.6% (196/205)
Antimicrobial choice		
Cefazolin	97.7% (176/180)	99.5% (195/196)
Vancomycin (for patients with immediate hypersensitivity to penicillin)	0% (0/4)^a^	0% (0/1)^a^
Dose (of cefazolin)		
2 g or as appropriate^b^	80.6% (145/180)	83.2% (163/196)
Preoperative timing of administration		
Within 60 min prior to incision (for cefazolin) or within 120 min prior to incision (for vancomycin)	Not available	Not available
Route		
IV	100% (180/180)	100% (196/196)
Intraoperative drug administration if duration of procedure ≥ 240 min	0% (0/2)	100% (2/2)
Duration of prophylaxis		
Should **not exceed 24 h** from time of induction (version 15)	78.3% (141/180)	–
Patients should receive only a **single preoperative dose** (version 16)	–	20.4% (40/196)

^a^Patients received clindamycin instead of vancomycin

^b^Appropriate dose based on age, weight, or renal function

### Timing of administration

Whilst preoperative timing of antibiotic administration was recorded on the anaesthetic record sheet during both audit periods, the time of incision was not noted, making it impossible to assess whether antibiotics were given at the appropriate time preoperatively.

### Duration of prophylaxis

Variable prophylaxis durations were observed in both audit periods. For the 2018 audit period, prophylaxis duration ranged from a single dose only at induction to 79.5 h after induction. Total duration of prophylaxis could only be determined for 180 patients. Over three-quarters of patients (78.5%) had antibiotics administered for 24 h or less, with an extended duration of prophylaxis (> 24 h) observed in 21.5% of patients. A shorter duration of prophylaxis was observed in the 2019 audit period, ranging from a single dose at induction to 50.5 h post-induction. Of the 196 patients in which the duration of prophylaxis could be determined, the majority (68.4%) had antibiotics administered for 24 h or less, with an extended duration of prophylaxis (> 24 h) observed in 11.2% of patients. However, only 20.4% of patients received single dose prophylaxis as per guideline recommendations.

### Surgical site infections

Very few patients developed an SSI within 90 days of procedure. From the 141 patients who were subsequently reviewed at Northern Health’s outpatient department for the 2018 audit period, only 2 patients exhibited symptoms of an SSI (one superficial SSI and one deep SSI). For the patient who developed a superficial SSI, management comprised of oral cefalexin (500 mg QID) for 10 days. For the deep SSI, management included IV cefazolin (2 g TDS), debridement and a skin graft. Of the 159 patients subsequently reviewed at Northern Health’s outpatient department for the 2019 audit period, no patients developed an SSI.

### Univariate analysis

Results of the univariate analysis for adherence to dose and duration can be found in Additional file 1: Tables S1, S2. The results of the overall adherence to guideline recommendations is highlighted in Additional file 1: Table S3. Age group was identified as a positive predictor of adherence to dose in both the 2018 and 2019 audit periods. The following variables were significantly associated with decreased adherence to dose recommendations in the 2019 audit period: female gender (OR: 0.30, 95% CI 0.11–0.86, p-value: 0.024); ASA score of 3 (OR: 0.06, 95% CI 0.007–0.47, p-value: 0.008); lower limb fractures (OR: 0.21, 95% CI 0.05–0.91, p-value: 0.038) and emergency admissions (OR: 0.24, 95% CI 0.07–0.85, p-value: 0.027).

In terms of correct adherence to duration of SAP (Additional file 1: Table S2), only the patient age variable was significantly associated with increased adherence between the two audit periods. Presenting with an ASA score ≥ 3, lower limb fractures, emergency procedures, diabetes as a comorbidity and a LOS of > 3 days was associated with decreased adherence to correct SAP duration across both audit periods.

All variables but gender were significantly associated with overall adherence to guidelines (Additional file 1: Table S3). Patient age group was the only variable across both audit periods that was significantly associated with increased adherence to guidelines, with the remaining variables being associated with decreased overall adherence to guidelines.

### Multivariable analysis

Results from the multivariable analysis shows that age group is a significant predictor of dose compliance for the 2018 study period (Additional file 1: Table S1). The following age groups were significantly associated with increased adherence to correct dose as per guidelines when compared to the reference category: 41–60 years (OR: 15.27, 95% CI 1.52–153.73, p-value: 0.021) and 61–80 years (OR: 7.67, 95% CI 1.66–35.38, p-value: 0.009). Similarly, for the 2019 study period, the same age groups were identified as being significantly associated with increased adherence to dose recommendations (41–60 years, OR: 12.16, 95% CI 1.12–131.82, p-value: 0.04 and 61–80 years, OR: 7.09, 95% CI 1.69–29.81, p-value: 0.007).

In terms of duration (Additional file 1: Table S2), for the 2018 study period, a LOS of more than 3 days was significantly associated with decreased adherence to the recommended duration (OR: 0.21, 95% CI 0.05–0.89, p-value: 0.034). Interestingly, for the 2019 study period, only limb fracture site was significantly associated with whether or not duration of SAP would be adhered to. Lower limb fractures were significantly associated with decreased adherence to duration (OR: 0.27, 95% CI 0.11–0.68, p-value: 0.005), indicating that postoperative antibiotic doses were more likely administered to those with lower limb fractures.

Whilst it was observed that younger patients (between the ages of 21–60 years) were more likely not to have postoperative antibiotics prescribed, there was no significant association detected in the multivariable analysis between age group and whether duration was compliant.

A significant association was observed between year of audit (or version of guideline used) and whether duration was compliant with guidelines (Χ^2^ (1) = 126.12, p value: < 0.001). Overall adherence to guidelines was also significantly associated with version 15 of the *Therapeutic Guidelines: Antibiotic* (Χ^2^ (1) = 84.88, p value: < 0.001). For the 2018 audit period, results from the multivariable analysis (Additional file 1: Table S3) indicate that diabetes as a comorbidity is significantly associated with a decreased overall adherence to guideline recommendations (OR: 0.26, 95% CI 0.09–0.75, p-value: 0.012). For the 2019 audit period, only limb fracture site was significantly associated with overall adherence to guideline recommendations (Additional file 1: Table S3). Lower limb fractures were associated with decreased overall adherence to guidelines (OR: 0.31, 95% CI 0.12–0.82, p-value: 0.018).

## Discussion

This is the first study, to our knowledge, to determine the prescribing practice of SAP for ORIF of closed fractures in Australia prior to, and following, an update to *Therapeutic Guidelines: Antibiotic*. Our study showed that overall adherence to the updated guidelines (version 16) was reduced in the second audit compared with adherence to version 15 in the first audit, and that duration of prophylaxis across both audit periods more closely matched version 15 guidelines. The latter finding may be attributed to the low number of patients (20.4%) receiving a single preoperative dose (in accordance with updated guidelines) in the second audit.

The administration of SAP is crucial in reducing the risk of SSI development, with SAP administration prior to incision an important factor [10]. Timing of SAP administration is related to SSI development, hence the importance of ensuring appropriate administration of preoperative antibiotics [13]. Administration of SAP after incision results in a significant SSI risk as compared to prior to incision [13]. Whilst there is no consensus on the optimal timing of administration, it is known that administration of SAP within 120 min prior to incision can reduce the risk of SSI development [13]. Current recommendations specify that antibiotics should be administered within 60 min before incision for most antibiotics (such as those with a short half-life) and up to 120 min before incision for antibiotics with a longer administration time, such as vancomycin [8, 13, 22].

We were unable to ascertain whether timing of preoperative administration was appropriate for any of our cases as the time of incision was not recorded. Similar results have been identified in the Surgical National Antimicrobial Prescribing Survey which showed suboptimal documentation of incision timing across Australian hospitals [4, 5]. Likewise, the timing of intraoperative SAP administration should also be considered. Intraoperative administration is recommended when a patient loses more than 1.5 L of blood or if the duration of a procedure exceeds two half-lives of the administered antimicrobial [8, 22]. We found that four patients underwent lengthy procedures where an intraoperative dose was required. Only the two patients in the second audit had antibiotics administered intraoperatively. Maintaining adequate serum and tissue concentrations of antimicrobials is required in order to minimise the risk of SSI development [8].

Growing evidence suggests that postoperative antibiotics are not required for many procedures, including ORIFs [13, 23]. While majority of cases in our audit had antibiotics administered within a 24 h period, between 11 and 21% of patients had an extended duration of prophylaxis (beyond 24 h). The reasons for this remain unclear, but are potentially attributable to ambiguous postoperative instructions, delays in nursing administration and orders that are not ceased in a timely fashion. A recent, large, multicentre retrospective audit has demonstrated that prolonging SAP beyond 24 h has little effect on reducing SSIs but rather increases the risk of *Clostridioides difficile* infection and acute kidney injury [24]. The use of excessive postoperative antibiotics can also result in increased adverse events such as rash, pruritus and gastrointestinal disturbances [1]. Results from the multivariable analysis in this study indicate that presenting with lower limb fractures was associated with patients receiving postoperative antibiotics. Of note, slightly higher rates of SSIs have been reported for ORIFs of lower extremity fractures [25]; this may be a reason why longer duration of prophylaxis is observed in such patients.

The majority of patients in this study received cefazolin as the agent of choice as per guideline recommendations, however, clindamycin was used as an alternative in penicillin-sensitive patients. Whilst vancomycin is listed in the *Therapeutic Guidelines: Antibiotic* as the preferred antimicrobial for patients with penicillin or beta lactam allergies, some international guidelines mention that clindamycin can also be used as an alternative agent [8]. Clindamycin may be used preferentially by surgeons over vancomycin due to the longer infusion time required to administer vancomycin as well as the potential for infusion site reactions such as red-man syndrome.

Although overall compliance with guidelines decreased following guideline update, individual compliance for drug choice and route of administration remained high. Similarly, while variations were observed in the doses administered, most doses were considered appropriate when patient factors such as age and renal function were taken into consideration. Up to 24 h of prophylactic antibiotic use was commonly observed, with fewer patients receiving prolonged SAP in the second audit. Furthermore, surgeon instructions in the second audit were more consistent and clearer than the first, with 10% of patients having instructions listed as ‘no antibiotics required’, aligning with new guideline recommendations. This instruction was not observed in the first audit, highlighting the gradual change in prescribing practice.

Adherence rates to guideline recommendations has been shown to improve following guideline revision. A study reviewing adherence to updated guidelines on the treatment of *C. difficile* infections in the United States found a significant increase in the prescribing of vancomycin and fidaxomicin in comparison to metronidazole as per guideline recommendations [26]. Likewise, a study conducted in Western Australia examined guideline adherence to the *Therapeutic Guidelines* for breast surgery and found that adherence greatly improved post guideline update from 13.3 to 49.2% [27].

Whilst it is unclear how the respective guidelines were distributed to improve adherence, it must be noted that dissemination of guidelines alone is insufficient to change practice. A systematic review has identified that simple dissemination of guidelines is insufficient to ensure adherence, thus the need to employ theory based interventions when trying to change practice [28].

This audit has shown that guideline amendment, in isolation, does not lead to a significant improvement in prescribing practice, thus greater efforts are required to ensure guidelines are appropriately disseminated to key stakeholders following revision. Our results also indicate that whilst age is a positive predictor of guideline adherence, other patient factors such as gender, ASA score or limb fracture site are not associated with greater adherence, hence the presence of additional influential factors that impact SAP decision making. There is a need to understand the barriers and enablers in a local setting that may impact the adherence rates to SAP guidelines. This can be achieved through the use of qualitative research methods such as interviews with key users of SAP guidelines such as orthopaedic surgeons, anaesthetists, pharmacists and nurses.

Limitations of this study include the small sample size that was audited at a single centre in Melbourne, thus results may not be generalizable or indicate guideline adherence for closed fracture ORIF procedures across the country. Furthermore, as only a few SSIs were detected in this study, we could not determine whether adherence to guidelines impacted SSI development. Timing of antibiotic administration in relation to incision time could not be determined, thus had to be excluded from the overall assessment of compliance with the *Therapeutic Guidelines: Antibiotic*. The 2019 audit also examines procedures 3 months after new guidelines were published. Given the proximity of the audit to the guideline release date, overall adherence with version 16 of the guidelines may have been impacted due to potentially incomplete dissemination, thus the small number of patients receiving single dose prophylaxis. Another potential contributor is the fact that the *Therapeutic Guidelines* is written primarily for general practitioners and trainee physicians [21], thus may not be readily accessed by senior surgical staff, resulting in suboptimal adherence to guidelines.

## Conclusion

This audit has shown that SAP prescribing for ORIF of closed fractures is relatively consistent for drug choice and route of administration with variations in dosing and duration of prophylaxis. Guideline adherence was greater with version 15 of the guidelines as compared to the updated version (v16), with the results of the regression analysis highlighting that patient specific factors contribute little to guideline adherence. Guidelines should be widely disseminated following updates to ensure that new knowledge is transferred to key stakeholders and should be coupled with other interventions that work in the local setting to ensure appropriate uptake. Further research is also required to understand why prophylaxis duration is prolonged in the orthopaedic setting and how prescribing practice can move towards single dose prophylaxis.

## Supplementary Information

**Additional file 1.** Univariate and multivariable analysis of adherence to dose, duration and overall recommendations in the *Therapeutic Guidelines: Antibiotic*.

## Data Availability

All data generated or analysed during this study are included in this published article [and its additional information files].

## Abbreviations

ASA: American Society of Anesthesiologists
BMI: Body mass index
CDC: Centers for Disease Control and Prevention
IV: Intravenous
LOS: Length of stay
MRSA: Methicillin-resistant *Staphylococcus aureus*
OR: Odds ratio
ORIF: Open reduction internal fixation
SAP: Surgical antimicrobial prophylaxis
SSI: Surgical site infection
WHO: World Health Organisation
