# Supplementary material for: Surgical antimicrobial prophylaxis in open reduction internal fixation procedures at a metropolitan hospital in Australia: a retrospective audit
Source: BMC Surg. 2021 Nov 23;21:404. doi: 10.1186/s12893-021-01398-7 (PMC8609780; doi:10.1186/s12893-021-01398-7)
Supplement: Supplementary file 1 — Additional file 1. Univariate and multivariable analysis of adherence to dose, duration and overall recommendations in the Therapeutic Guidelines: Antibiotic. [file 12893_2021_1398_MOESM1_ESM.docx]

**Table S1. Adherence to dose listed in *Therapeutic Guidelines: Antibiotic* using univariate and multivariable analysis**

| **Variables** | | **Adherence to dose  v15 (2018)** | | | | **Adherence to dose v16 (2019)** | | | |
| --- | --- | --- | --- | --- | --- | --- | --- | --- | --- |
|  |  | **Univariate analysis** | | **Multivariable analysis** | | **Univariate analysis** | | **Multivariable analysis** | |
|  |  | **Odds ratio [95% CI]** | **p-value** | **Odds ratio [95% CI]** | **p-value** | **Odds ratio [95% CI]** | **p-value** | **Odds ratio [95% CI]** | **p-value** |
| **Gender** | |  | |  | |  | |  | |
|  | Male | Reference | Reference | Reference | Reference | Reference | Reference | Reference | Reference |
|  | Female | 0.50 [0.21 - 1.23] | 0.132 | 0.63 [0.22 - 1.82] | 0.39 | 0.30 [0.11 - 0.86] | 0.024 | 0.52 [0.16 - 1.74] | 0.29 |
| **Patient age** | |  | |  | |  | |  | |
|  | 0-20 years | 1.87 [0.60 - 5.83] | 0.283 | 2.84 [0.38 - 20.97] | 0.307 | 4.87 [0.97 - 24.44] | 0.054 | 0.57 [0.04 - 7.84] | 0.675 |
|  | 21-40 years | 9.33 [1.15 - 75.52] | 0.036 | 14.31 [0.93 - 219.28] | 0.056 | 13.09 [2.73 - 62.64] | 0.001 | 3.80 [0.49 - 29.13] | 0.2 |
|  | 41-60 years | 12.44 [1.56 - 99.59] | 0.017 | 15.27 [1.52 -153.73] | 0.021 | 24.96 [3.08 - 202.18] | 0.003 | 12.16 [1.12 - 131.82] | 0.04 |
|  | 61-80 years | 5.78 [1.55 - 21.49] | 0.009 | 7.67 [1.66 -35.38] | 0.009 | 7.91 [2.05 - 30.50] | 0.003 | 7.09 [1.69 - 29.81] | 0.007 |
|  | >80 years | Reference | Reference | Reference | Reference | Reference | Reference | Reference | Reference |
| **ASA score** | |  | |  | |  | |  | |
|  | 1 | Reference | Reference | Reference | Reference | Reference | Reference | Reference | Reference |
|  | 2 | 1.42 [0.4338 - 4.60] | 0.559 | 2.07 [0.37 - 11.57] | 0.407 | 0.26 [0.03 - 2.35] | 0.233 | 0.14 [0.01 - 1.88] | 0.138 |
|  | 3 | 0.80 [0.25 - 2.55] | 0.702 | 3.81 [0.51 - 28.51] | 0.192 | 0.06 [0.007 - 0.47] | 0.008 | 0.06 [0.004 - 1.11] | 0.059 |
|  | 4 | 0.47 [0.07 - 3.00] | 0.424 | 2.78 [0.20 - 38.50] | 0.446 | PP^c^ | 0.999 | PP^c^ | 0.999 |
|  | 5 | NA^a^ | NA^a^ | NA^a^ | NA^a^ | Excluded^b^ | Excluded^b^ | Excluded^b^ | Excluded^b^ |
|  | Not recorded | 1.31 [0.30 - 5.83] | 0.721 | 4.64 [0.61 - 35.59] | 0.14 | 0.16 [0.015 - 1.59] | 0.116 | 0.08 [0.004 - 1.58] | 0.096 |
| **Limb fracture site** | |  | |  | |  | |  |  |
|  | Upper limb | Reference | Reference | Reference | Reference | Reference | Reference | Reference | Reference |
|  | Lower limb | 0.44 [0.16 - 1.23] | 0.118 | 1.29 [0.32 - 5.13] | 0.722 | 0.21 [0.05 - 0.91] | 0.038 | 0.83 [0.13 - 5.50] | 0.848 |
| **Type of admission** | |  | |  | |  | |  | |
|  | Elective | Reference | Reference | Reference | Reference | Reference | Reference | Reference | Reference |
|  | Emergency | 0.38 [0.14 - 1.06] | 0.065 | 0.66 [0.18 - 2.40] | 0.525 | 0.24 [0.07 - 0.85] | 0.027 | 0.55 [0.11 - 2.72] | 0.46 |
| **Diabetes status** | |  | |  | |  | |  | |
|  | Yes | 0.46 [0.17 - 1.23] | 0.121 | 0.43 [0.12 - 1.54] | 0.194 | 0.45 [0.15 - 1.37] | 0.16 | 0.98 [0.25 - 3.78] | 0.972 |
|  | No | Reference | Reference | Reference | Reference | Reference | Reference | Reference | Reference |

^a:^  No patients with an ASA score of 5 in 2018 audit; ^b:^  Excluded due to low patient numbers; ^c:^ Perfect prediction (OR not valid)

Abbreviations: ASA - American Society of Anesthesiologists score

**Table S2. Adherence to duration listed in *Therapeutic Guidelines: Antibiotic* using univariate and multivariable analysis**

| **Variables** | | **Adherence to duration v15 (2018)** | | | | **Adherence to duration v16 (2019)** | | | |
| --- | --- | --- | --- | --- | --- | --- | --- | --- | --- |
|  |  | **Univariate analysis** | | **Multivariable analysis** | | **Univariate analysis** | | **Multivariable analysis** | |
|  |  | **Odds ratio [95% CI]** | **p-value** | **Odds ratio [95% CI]** | **p-value** | **Odds ratio [95% CI]** | **p-value** | **Odds ratio [95% CI]** | **p-value** |
| **Gender** | |  | |  | |  | |  | |
|  | Male | Reference | Reference | Reference | Reference | Reference | Reference | Reference | Reference |
|  | Female | 1.03 [0.51 - 2.10] | 0.932 | 2.06 [0.82 - 5.19] | 0.125 | 0.80 [0.40 - 1.61] | 0.535 | 1.79 [0.74 - 4.31] | 0.196 |
| **Patient age** | |  | |  | |  | |  | |
|  | 0-20 years | 5.56 [1.17 -26.33] | 0.031 | 2.70 [0.29 - 25.20] | 0.384 | 9.6 [0.99 - 93.16] | 0.051 | 4.72 [0.28 - 78.34] | 0.279 |
|  | 21-40 years | PP^c^ | 0.998 | PP^c^ | 0.998 | 21.38 [2.71 - 168.80] | 0.004 | 6.90 [0.56 - 85.36] | 0.132 |
|  | 41-60 years | 4.45 [1.10 - 15.66] | 0.036 | 1.81 [0.38 - 8.75] | 0.459 | 14.18 [1.76 - 114.44] | 0.013 | 4.02 [0.33 - 49.42] | 0.277 |
|  | 61-80 years | 0.69 [0.30 - 1.59] | 0.39 | 0.45 [0.16 - 1.27] | 0.131 | 2.77 [0.28 - 27.84] | 0.387 | 0.87 [0.07 - 11.46] | 0.918 |
|  | >80 years | Reference | Reference | Reference | Reference | Reference | Reference | Reference | Reference |
| **ASA score** | |  | |  | |  | |  | |
|  | 1 | Reference | Reference | Reference | Reference | Reference | Reference | Reference | Reference |
|  | 2 | 0.44 [0.13 - 1.46] | 0.18 | 2.25 [0.39 - 12.85] | 0.362 | 0.96 [0.41 - 2.27] | 0.926 | 1.21 [0.42 - 3.51] | 0.73 |
|  | 3 | 0.22 [0.06 - 0.73] | 0.013 | 2.43 [0.37 - 16.18] | 0.358 | 0.28 [0.08 - 0.94] | 0.04 | 1.75 [0.33 - 9.43] | 0.515 |
|  | 4 | 0.08 [0.01 - 0.50] | 0.007 | 0.87 [0.08 - 9.51] | 0.911 | PP^c^ | 0.999 | PP^c^ | 0.999 |
|  | 5 | NA^a^ | NA^a^ | NA^a^ | NA^a^ | Excluded^b^ | Excluded^b^ | Excluded^b^ | Excluded^b^ |
|  | Not recorded | 0.54 [0.12 - 2.40] | 0.418 | 2.68 [0.35 - 20.73] | 0.345 | 1.41 [0.49 - 4.09] | 0.525 | 2.44 [0.64 - 9.24] | 0.191 |
| **Limb fracture site** | |  | |  | |  | |  | |
|  | Upper limb | Reference | Reference | Reference | Reference | Reference | Reference | Reference | Reference |
|  | Lower limb | 0.13 [0.04 - 0.44] | 0.001 | 0.69 [0.13 - 3.61] | 0.664 | 0.13 [0.06 - 0.28] | <0.001 | 0.27 [0.11 - 0.68] | 0.005 |
| **Type of admission** | |  | |  | |  | |  | |
|  | Elective | Reference | Reference | Reference | Reference | Reference | Reference | Reference | Reference |
|  | Emergency | 0.11 [0.03 - 0.38] | <0.001 | 0.34 [0.08 - 1.49] | 0.15 | 0.17 [0.07 - 0.35] | <0.001 | 0.47 [0.19 - 1.17] | 0.103 |
| **Diabetes status** | |  | |  | |  | |  | |
|  | Yes | 0.24 [0.10 - 0.56] | 0.001 | 0.54 [0.19 - 1.56] | 0.258 | 0.32 [0.07 - 1.41] | 0.132 | 1.54 [0.24 - 9.78] | 0.65 |
|  | No | Reference | Reference | Reference | Reference | Reference | Reference | Reference | Reference |
| **LOS** | |  | |  | |  | |  | |
|  | 0-3 days | Reference | Reference | Reference | Reference | Reference | Reference | Reference | Reference |
|  | >3 days | 0.08 [0.03 - 0.22] | <0.001 | 0.21 [0.05 - 0.89] | 0.034 | 0.15 [0.06 - 0.38] | <0.001 | 0.51 [0.15 - 1.77] | 0.29 |

^a:^  No patients with an ASA score of 5 in 2018 audit; ^b:^  Excluded due to low patient numbers; ^c:^ Perfect prediction (OR not valid)

Abbreviations: ASA - American Society of Anesthesiologists score; LOS – Length of stay

**Table S3. Overall adherence to recommendations in *Therapeutic Guidelines: Antibiotic* using univariate and multivariable analysis**

| **Variables** | | **Overall adherence to guidelines v15 (2018)** | | | | **Overall adherence to guidelines v16 (2019)** | | | |
| --- | --- | --- | --- | --- | --- | --- | --- | --- | --- |
|  |  | **Univariate analysis** | | **Multivariable analysis** | | **Univariate analysis** | | **Multivariable analysis** | |
|  |  | **Odds ratio [95% CI]** | **p-value** | **Odds ratio [95% CI]** | **p-value** | **Odds ratio [95% CI]** | **p-value** | **Odds ratio [95% CI]** | **p-value** |
| **Gender** | |  | |  | |  | |  | |
|  | Male | Reference | Reference | Reference | Reference | Reference | Reference | Reference | Reference |
|  | Female | 0.896 [0.47 - 1.72] | 0.741 | 1.41 [0.61 - 3.26] | 0.428 | 0.72 [0.35 - 1.48] | 0.373 | 1.78 [0.70 - 4.50] | 0.225 |
| **Patient age** | |  | |  | |  | |  | |
|  | 0-20 years | 2.51 [0.90 - 6.99] | 0.078 | 0.95 [0.16 - 5.58] | 0.955 | 10.29 [1.01 - 100.22] | 0.045 | 3.89 [0.22 - 68.69] | 0.354 |
|  | 21-40 years | 19.44 [2.43 - 155.42] | 0.005 | 4.72 [0.36 - 62.71] | 0.24 | 21.86 [2.74 - 174.32] | 0.004 | 6.07 [0.46 - 80.51] | 0.171 |
|  | 41-60 years | 8.03 [ 2.16 - 29.83] | 0.002 | 4.16 [0.88 - 19.64] | 0.072 | 16.14 [1.99 - 130.72] | 0.009 | 3.98 [0.31 - 51.57] | 0.291 |
|  | 61-80 years | 1.24 [ 0.55 - 2.80] | 0.614 | 1.05 [0.38 - 2.90] | 0.924 | 2.77 [0.28 - 27.84] | 0.387 | 0.77 [0.06- 10.57] | 0.848 |
|  | >80 years | Reference | Reference | Reference | Reference | Reference | Reference | Reference | Reference |
| **ASA score** | |  | |  | |  | |  | |
|  | 1 | Reference | Reference | Reference | Reference | Reference | Reference | Reference | Reference |
|  | 2 | 0.73 [0.28 - 1.93] | 0.53 | 1.52 [0.37 - 6.24] | 0.564 | 0.85 [0.35 - 2.06] | 0.72 | 1.20 [0.40 - 3.62] | 0.743 |
|  | 3 | 0.27 [0.10 - 0.72] | 0.009 | 1.53 [0.31 - 7.52] | 0.596 | 0.25 [0.08 - 0.86] | 0.028 | 1.76 [0.31 - 9.95] | 0.525 |
|  | 4 | 0.20 [0.04 - 1.08] | 0.062 | 1.40 [0.17 - 11.63] | 0.756 | PP^c^ | 0.999 | PP^c^ | 0.999 |
|  | 5 | NA^a^ | NA^a^ | NA^a^ | NA^a^ | Excluded^b^ | Excluded^b^ | Excluded^b^ | Excluded^b^ |
|  | Not recorded | 1.01 [ 0.29 - 3.56] | 0.984 | 2.93 [0.55 - 15.51] | 0.207 | 1.17 [0.39 - 3.54] | 0.785 | 2.21 [0.53 - 9.15] | 0.275 |
| **Limb fracture site** | |  | |  | |  | |  | |
|  | Upper limb | Reference | Reference | Reference | Reference | Reference | Reference | Reference | Reference |
|  | Lower limb | 0.21 [0.09 - 0.50] | <0.001 | 0.79 [0.23 - 2.68] | 0.705 | 0.13 [0.06 - 0.29] | <0.001 | 0.31 [0.12 -0.82] | 0.018 |
| **Type of admission** | |  | |  | |  | |  | |
|  | Elective | Reference | Reference | Reference | Reference | Reference | Reference | Reference | Reference |
|  | Emergency | 0.17 [0.07 - 0.42] | <0.001 | 0.48 [0.16 - 1.44] | 0.192 | 0.13 [0.06 - 0.29] | <0.001 | 0.41 [0.16 - 1.08] | 0.071 |
| **Diabetes status** | |  | |  | |  | |  | |
|  | Yes | 0.15 [0.06 - 0.37] | <0.001 | 0.26 [0.09 - 0.75] | 0.012 | 0.31 [0.07 - 1.40] | 0.128 | 1.53 [0.24 - 9.87] | 0.657 |
|  | No | Reference | Reference | Reference | Reference | Reference | Reference | Reference | Reference |
| **LOS** | |  | |  | |  | |  | |
|  | 0-3 days | Reference | Reference | Reference | Reference | Reference | Reference | Reference | Reference |
|  | >3 days | 0.14 [0.07 - 0.31] | <0.001 | 0.34 [0.09 - 1.27] | 0.107 | 0.12 [0.04 -0.32] | <0.001 | 0.41 [0.11 - 1.54] | 0.187 |

^a:^  No patients with an ASA score of 5 in 2018 audit; ^b:^  Excluded due to low patient numbers; ^c:^ Perfect prediction (OR not valid)

Abbreviations: ASA - American Society of Anesthesiologists score; LOS – Length of stay
